# Supplementary material for: Physicochemical Properties and Effects of Fruit Pulps from the Amazon Biome on Physiological Parameters in Rats
Source: Nutrients. 2021 Apr 28;13(5):1484. doi: 10.3390/nu13051484 (PMC8146226; doi:10.3390/nu13051484)
Supplement: Supplementary file 1 [file nutrients-13-01484-s001.zip › nutrients-1173463-supplementary.pdf]

Tabela S1. Biochemical results after Amazonian pulp fruits consumption in Wistar rats (n=10 rats/group).

Experimental Group	Hematocrit (%)	Leukocytes (mm^3)	Blood glucose (mg/dL)	Cholesterol (mg/dL)	Triacylglycerols (mg/dL)	TGO (U/I)	TGP (U/I)	Urea (mg/dL)	Creatine (mg/dL)
T1- Control	0,42^{a}±0,03	7670^{a}±1381,9	97^{a}±20,1	108,9^{a}±7,8	61,4^{a}±9,3	98^{a,b}±18,6	39,7^{a}±7,1	25,2^{a}±5,7	0,26^{a}±0,04
T2 – Araçá-boi (*Eugenia stipitata*)	0,45^{a}±0,01	6930^{a}±506,2	96^{a}±11,2	101,3^{a}±22,7	74,6^{a,b}±11,6	82^{a}±4,6	36,7^{a}±4,3	23,1^{a}±1,8	0,28^{a}±0,02
T3 – Abiu (*Pouteria caimito*)	0,43^{a}±0,01	4167^{b}±1410,9	96^{a}±11,2	103,2^{a}±15,6	74,3^{a,b}±22,8	85^{a,b}±8,5	38,1^{a}±3,2	22,1^{a}±1,5	0,30^{a}±0,06
T4 – Araticum (*Annona crassiflora*)	0,46^{a}±0,02	4365^{b}±1186,3	88^{a}±28,5	119^{a}±17,0	99,5^{b}±36,1	91^{a,b}±14,4	40,6^{a}±5,8	23,3^{a}±2,4	0,28^{a}±0,02
T5 – Biri-biri (*Averrhoa bilimbi* L.)	0,44^{a}±0,03	7025^{a}±1103,1	90^{a}±14,62	105,8^{a}±11,9	78,5^{a,b}±27,8	116^{b}±26,1	37,7^{a}±4,5	28,17^{a}±2,6	0,27^{a}±0,03
T6 – Yellow Mangosteen (*Garcinia xanthochymus*)	0,43^{a}±0,01	6205^{a}±779,0	99^{a}±10,7	104,9^{a}±18,4	86,2^{a,b}±32,5	81^{a}±9,9	36,8^{a}±4,8	22,3^{a}±2,6	0,28^{a}±0,04

[1]Mean ± standard deviation. n = 10. Means followed by the same letters in a column do not differ significantly (p≤0.05) by the one-way ANOVA test followed by post-hoc Tukey test or Kruskal Wallis test.
